# Geographic Distribution of Clinical Trials for Advanced-Stage Cancer

**DOI:** 10.1001/jamaoncol.2024.1690

**Published:** 2024-06-03

**Authors:** Wade T. Swenson, Abigail Swenson, Emily Westergard, Zachary Schroeder

**Affiliations:** 1 Lakewood Cancer Center, Lakewood Health System, Staples, Minnesota; 2 Department of Internal Medicine, University of North Dakota School of Medicine, Fargo; 3 Medical College of Wisconsin, Green Bay, Wisconsin; 4 Gundersen Health System, La Crosse, Wisconsin; 5 currently a medical student at University of Kansas School of Medicine, Wichita

## Abstract

This quality improvement study evaluates the geographic distribution of clinical trials and assesses the distances patients with cancer must travel to access a clinical trial site.

Clinical trials play a crucial role in advancing cancer research and treatment. The National Cancer Comprehensive Network Guidelines state, “The best management of any patient with cancer is in a clinical trial. Participation in clinical trials is especially encouraged.”^1^ Despite the potential benefits of participating in a clinical trial, there can be significant burdens for both clinicians and patients in accessing them, such as geographic distance from trial sites and lack of awareness about available trials.^2^ Health disparities in access to clinical trials have been widely recognized, particularly in rural populations and among racial and ethnic minority groups.^3^

A 2015 study of the geographic distribution of clinical trials^4^ reported that 45.6% of patients with metastatic breast cancer, 50.2% with prostate cancer, 52.2% with colorectal cancer, and 38.4% with non–small cell lung cancer would need to drive more than 60 minutes 1 way to access a clinical trial site. The present study updates and expands these data.

## Methods

This quality improvement study followed the SQUIRE reporting guideline. Ethics review and informed consent were waived because this study was not considered human participants research. The ClinicalTrials.gov database of clinical trials^5^ was accessed on November 25, 2022, to identify interventional clinical trials actively recruiting patients for diagnoses of metastatic breast, colon, lung, pancreatic, and prostate cancers. After identifying unique zip codes for clinical trials, Maptitude geographic information system software (Caliper Corporation) was used to calculate the population living within 30, 60, and 120 miles of a clinical trial site using 2020 US Census data. The data were further stratified by urban or rural status, race and ethnicity, and other socioeconomic measures to assess disparities in access to clinical trials.

## Results

An analysis of 701 clinical trials found that the current clinical trial infrastructure provides access for most US residents diagnosed with common advanced-stage cancers. Most of the US population lives within 30 miles of a clinical trial site, which is considered a reasonable distance for patients to travel for treatment and clinical trial participation.

Access to clinical trials varied by cancer type and racial and ethnic group. The percentage of the US population living within 30 miles of a clinical trial site varied based on cancer diagnosis (breast, 81.8%; colon, 62.9%; lung, 82.0%; pancreatic, 65.7%; and prostate, 81.5%). Racial disparities were also identified, with American Indian and Alaska Native patients consistently having reduced access to clinical trials (eg, 42.1%-60.8% <30 miles from a site) compared with patients from other racial and ethnic groups (eg, 84.7%-95.1% of Asian patients <30 miles from a site) (Table).

**Table.** Percentage of US Population Segments Residing Within Distance Categories of Active Clinical Trial Sites for Common Advanced Cancers

Cancer type by distance to trial site, miles	Population segment, %								
	All	Aged >65 y	Household income <$25 000	Race and ethnicity					
				American Indian or Alaska Native	Asian	Black	Hispanic	White
**Breast cancer**									
<30	81.8	79.2	75.0	56.5	95.0	84.5	82.2	80.0
<60	93.3	92.5	89.5	78.4	98.5	94.8	89.8	92.5
<120	98.1	97.9	96.1	92.1	99.7	98.9	93.7	97.9
**Colon cancer**									
<30	62.9	58.6	54.7	42.1	86.7	67.5	67.2	59.2
<60	79.0	76.3	71.9	60.6	93.0	82.0	78.9	77.0
<120	93.5	93.2	90.3	75.1	98.2	96.2	86.3	93.1
**Lung cancer**									
<30	82.0	79.5	75.0	60.8	95.1	85.7	83.1	79.5
<60	93.1	92.3	89.3	76.9	98.1	94.5	89.9	92.3
<120	97.7	97.4	95.6	91.3	99.3	98.8	93.1	97.4
**Pancreatic cancer**									
<30	65.7	62.2	58.0	40.9	84.7	70.7	68.6	62.7
<60	81.4	80.0	75.6	57.9	90.9	83.0	79.0	80.5
<120	93.8	93.5	91.1	80.9	95.7	95.5	78.0	94.0
**Prostate cancer**									
<30	81.5	79.3	75.0	55.7	95.1	85.6	79.3	79.7
<60	92.9	92.4	89.2	74.5	98.0	95.6	85.3	92.5
<120	96.7	96.7	94.4	89.3	99.2	98.7	88.7	96.7

## Discussion

This quality improvement analysis of clinical trials for metastatic breast, lung, colon, pancreatic, and prostate cancers found that a large proportion of the US population lived within 30 miles of a clinical trial site. This finding suggests that while many clinical trials are available, they are not evenly distributed across the country and may not be accessible to all individuals, particularly racial and ethnic minority individuals. This disparity in access to clinical trials raises important questions about equity and fairness in the distribution of health care resources and opportunities for treatment.

Limitations of this study include addressing other barriers to clinical trial access, including a lack of awareness about available trials, concerns about the cost and adverse effects of experimental treatments, and eligibility criteria. Other cancer types and clinical trial phases may have different geographic distributions and accessibility patterns. To address these disparities and ensure equitable access to clinical trials, it will be important to continue to develop innovative solutions that make clinical trials more accessible to all patients, regardless of their location or racial and ethnic group.

## Notes

Supplement.
Data Sharing Statement
