# Supplementary material for: Geographic Distribution of Clinical Trials for Advanced-Stage Cancer
Source: JAMA Oncol. 2024 Jun 3;10(8):1132–3. doi: 10.1001/jamaoncol.2024.1690 (PMC11148782; doi:10.1001/jamaoncol.2024.1690)
Supplement: Supplement. — Data Sharing Statement [file jamaoncol-e241690-s001.pdf]

Data Sharing Statement

Swenson. Geographic Distribution of Clinical Trials for Advanced-Stage Cancer. *JAMA Oncol*. Published June 03, 2024. doi:10.1001/jamaoncol.2024.1690

Data
Data available: Yes
Data types: Data (not involving human participants)
How to access data: drswenson@lakewoodhealthsystem.com
When available: With publication

Supporting Documents
Document types: None

Additional Information
Who can access the data: Researchers whose proposed use of the data has been approved.
Types of analyses: Research
Mechanisms of data availability: Email
Any additional restrictions: None
